# Application of a hydrophobic coating to a pressurized pipe and its effect on energy losses and fluid flow profile

**DOI:** 10.1038/s41598-024-59075-7

**Published:** 2024-04-08

**Authors:** Antonio J. Muñóz, Juan Reca, Juan Martínez

**Affiliations:** 1 Department of Engineering, University of Almería, Ctra. Sacramento S.N. La Cañada de S. Urbano, 04129 Almería, Spain; 2 CIAIMBITAL Research Center (Center for Mediterranean Intensive Agro-Systems and Food Technology, University of Almería, Ctra. Sacramento S.N. La Cañada de S. Urbano, 04129 Almería, Spain

**Keywords:** Pressurized pipe flow, Drag reduction, Head loss reduction, Hydrophobic coating, Flow profile, Engineering, Physics

## Abstract

The use of additives, generally called DRAs (Drag Reducing Additives), has been proposed to re-duce the energy consumption in pressurized pipes. Although many research works have been conducted to analyze the effect of these additives, less attention have been devoted to the application of coatings to the pipe wall. This paper demonstrates that the application of a hydrophobic coating to the pipe can lead to a head loss reduction for a transition flow regime with moderate Reynolds number values (Re). For this purpose, an experiment was conducted to compare the performance of both coated and uncoated pipes by measuring the head losses and assessing the Drag Reduction Percentage (%DR) and the pipe friction factor (f). This was done for two Polyvinylchloride (PVC) pipes with different nominal diameters (PVC90 and PVC63). In addition, the flow velocity distribution was also measured in all these tests. The %DR decreased as the Re values increased, with the reduction being notably less pronounced for higher Re values. This could be explained by the fact that a partial slip condition is induced by the hydrophobic product. Its effect is significant for a transition regime where the effect of viscosity is important, but it becomes negligible for increasing levels of turbulence. No significant differences were observed in the flow distribution between coated and uncoated pipes, which seems to indicate that the velocity change could be limited to the near-wall viscous sublayer. The results of this work open an important research line aimed at reducing energy costs and the carbon footprint in pipe fluid distribution systems.

## Introduction

Pipelines have been used as an appropriate technical solution to transport fluids from one location to another—from the very first terracotta and copper pipes used by ancient civilizations to modern-day water, oil, and gas lines.

Although the technology and materials of pipelines have evolved and improved throughout history, the fundamentals of pressurized fluid flow have not been properly understood until more recently, thanks to the contribution of eminent researchers that have focused on the study of friction in pipes^1^.

As the use of piping systems became generalized, the concern about reducing the amount of energy required to transport fluids through pressurized pipes increased. More recently, efforts to reduce energy costs also involved environmental goals, such as reducing carbon footprints in fluid transport and, consequently, greenhouse gas (GHG) emissions.

Heuristic optimization methods have been effectively applied to pipe network design and management, successfully reducing energy costs. A wide array of optimization methods tackles this challenge. Within these approaches, both single-objective methods—like genetic algorithms or hybrid genetic/search space reduction—and multi-objective methods, including scatter search, stand out as notable solutions^2–4^.

One of the most immediate solutions to reduce energy losses in pipes is decreasing pipe roughness. The less rough the pipe is, the lower the friction factor and the less prone it is to generate high intensity turbulence. In many applications, such as irrigation, piping systems have evolved towards smoother materials, such as plastic pipes (PVC, PE). However, this strategy has a technical limit in hydraulically smooth pipes, which are pipes with negligible roughness.

To overcome this limitation, different techniques to control turbulence and reduce head losses were devised by fluid mechanics engineers and researchers. One of the most interesting control technique is the modification of the flow field by varying the pipe wall geometry with different flow control structures such as riblets, grooves, shark-skin surfaces, honeycombs and screens^5^. Following this approach, and moving a step forward, some researchers have realized that a suitable modification of the mean velocity profile can lead to a complete collapse of turbulence, causing a turbulent flow to fully relaminarize, thus considerably reducing head losses.

Different methods have been proposed to modify the velocity profile. Kühnen et al. analyzed three different devices to modify the flow profile: several small rotors mounted on a support structure within the pipe to introduce fluid perturbances, wall-normal and streamwise injection through an annular gap, and a moving pipe^6^. In another study, these researchers investigated two different devices: a stationary obstacle (inset) and a device injecting fluid through an annular gap close to the wall. These were utilized to modify the velocity profile by enhancing the flow in the center of the pipe while accelerating the flow in the near wall region^5^. These authors found total relaminarization up to Reynolds numbers of 6000, and a skin friction reduction by a factor of 3.4. In another research work, a briefly and rapidly shifted moving pipe segment in the streamwise direction was used to modify the flow velocity profile. The authors noted an exponential decrease in fluctuations and observed flow relaminarization at low to moderate flow speeds. Additionally, they determined that the minimum streamwise length for acceleration to be effective increased linearly with the Reynolds number^7^.

Another interesting technique devised to reduce turbulence and head losses involved, is incorporating additives to the fluid. These substances are generally called DRAs (Drag Reducing Additives). Drag reduction (DR) is defined as the ability to reduce the frictional resistance in turbulent flows in the pipeline, particularly using low concentrations of certain additives. These additives can be classified as follows: polymers, surfactants, fibers, micro-bubbles, and compliant coating^8^.

The first research works dealing with the effect of DRAs date back to the last century. In 1948, Toms^9^ discovered that the addition of a small amount of polymer into a turbulent Newtonian solvent (parts per million by weight), which results in a non-Newtonian fluid solution, can reduce the skin frictional drag on a stationary surface by up to 80%. Later, in 1967, Ram et al.^10^ investigated the effect of the addition of small quantities of soluble polymers, such as polyisobutylene, to crude oil and kerosine pipes. They found that the friction coefficient was markedly reduced in turbulent flows. Lumley^11,12^ discussed the drag reduction phenomenon theoretically, stating that it may be related to changes in the large eddy structure caused by the polymer induced changes in the small eddies. Other researchers demonstrated that the DRA affected the geometry and the structure of the turbulent boundary layer^13^. Yang and Dou^14^ found that the ratio of effective viscosity caused by polymers to kinematic viscosity of fluid was proportional to the Reynolds number and that the proportionality factor depends on polymer type and concentration.

The use of DRA agents is an active field of research^15,16^ and has become of paramount interest in many branches of industry, such as the transport of crude oil^17^. Recently, research lines have focused on the use of natural polymers in order to be more environmentally friendly^18^. For example, Edomwonyi-Otu et al. analyzed the effectiveness of natural polymers (biopolymers) as drag reducers in flows of oil–water mixtures. They studied two natural polymers, namely xanthan gum (XG) and guar gum (GG) The result showed that the gums (natural polymers) performed better as drag reducers in freshwater than in mixture with oil. Specifically, the drag reduction (DR) of 200 pm GG and XG solutions at a Reynolds number of 59,000 in freshwater was 39% and 44%, respectively, while with the addition of 50% oil fraction, it was reduced to 19% and 32%, respectively^19^. Other researchers analyzed the effect of flows of clay suspensions and they found that the resistance of the flow was considerably reduced for rough flow boundaries^20^.

Another important field of research is the study of the performance of the DRPs, not only in single phase flows but also in multiphase fluid flows^21,22^. Alsurkji et al.^8^ analyzed the effect of drag-reducing polymers (DRPs) for single water and air–water flow, and they compared the experimental results with simulated results obtained by a computational fluid dynamic (CFD) software. These authors found that an increase in DRP concentrations results in an increase in drag reduction of up to 45% in single-phase water flow and up to 42% in air–water stratified flow. This research team also investigated the effects of water-soluble polar ZETAG^®^ 8165 and nonpolar oil-soluble polyisobutylene (PIB) DRPs on drag reduction using two-phase air–water and air–oil flows, and three-phase air–oil–water flow. The authors found that the interaction between the DRP state (chemical structure and hydrodynamic size) and the external environment (fluid flow pattern, polarity, phase morphology, and intensity of turbulence) dictated its ability to dampen turbulent eddies, streamline the velocity field, and eventually increase the thickness of the laminar sublayer^23^.

An appropriate coating candidate substance is a hydrophobic product that repels water. Superhydrophobic surfaces have been investigated recently due to their potential for hydrodynamic frictional drag reduction The use of a pipe coating has both advantages and drawbacks. The main advantage is that the product can be added during the manufacturing process of the pipe, thereby avoiding the need to continuously inject a DRA substance. This could entail not only a cost reduction but also a reduction of residues, which is particularly interesting considering these additives may be hazardous for pipe systems that convey water or liquids used for human consumption. However, the major obstacle to the successful application of the coating in engineering applications is the durability and integrity of the coating without flakes of the hydrophobic substance being peeled off by the drag of the fluid flow. Some research works have focused on the durability of superhydrophobic surfaces in fully immersed conditions^24^.

While the application of a hydrophobic coating presents a simpler procedure than using complicated flow control structures, its utility may be somewhat constrained, as demonstrated in this work.

Although the mechanism affecting hydrophobic drag reduction is relatively well understood for laminar flows, research exploring this effect in fully turbulent flows has emerged more recently, driven by the need to enhance flow test technologies. A interesting review on hydrophobic drag reduction in fully developed turbulent flows is that by^25^. However, these compilations of studies exclusively focus on open flows, with significantly fewer investigations into the hydrophobic effect on pressurized pipes. Moreover, the research conducted has predominantly concentrated on drag reduction for laminar flows^26^. For example, Watanabe et al.^27^ reported that a 14% drag reduction was achieved for a laminar flow of water in a pipe with a highly water-repellent wall.

Consequently, the present paper aims to demonstrate that the application of a hydrophobic product in pipes can lead to a reduction in the friction of a fluid with the inner duct wall, as other previous studies have reported. However, in this research a wide range of Reynolds numbers have been covered within the transition region with the aim of extending this conclusion to a hydraulic regime insufficiently studied in previous works. Numerous studies attribute the hydrophobic effect to a slip condition that affects the velocity profile of the flow. In fact, some authors have proposed the approach of a “negative roughness” to model this slip condition^28^. This project conducted experimental tests to analyze drag reduction and possible changes in the flow distribution in the pipe. The results of this work open an important research line aimed at reducing energy costs and the carbon footprint in pipe fluid distribution systems.

## Material and methods

### Hydrophobic product

Hydrophobicity is the quality of certain materials to repel water. Water molecules interact with each other and with polar substances through hydrogen bonding and dipole–dipole interactions, which does not occur in hydrophobic substances. In the presence of these substances, the hydrogen bonds between the water molecules are maximized, giving rise to higher surface tension, thereby preventing the ability of water to wet.

This characteristic is measured by the contact angle caused by a drop lying on a surface. Hydrophobic substances are those with contact angles greater than 90°. If this angle is higher than 160º, the substance is called super hydrophobic.

For this research, a product called ULTRA EVER DRY was purchased online (https://www.ultraeverdry-store.eu/). This product is composed of a two-component coating that provides superhydrophobic and oleophobic effects, with contact angles between 160° and 175°, repelling most water-based and some oil-based liquids. The bottom coat bonds to most materials and acts as a primer. It provides a consistent material for the top coat to bond to while interacting with the top coat to self-assemble the surface, creating finely textured geometry. This surface is comprised of patterns of geometric shapes and billions of interstitial spaces. This product was chosen for its superhydrophobic effects and because it has vastly improved adhesion and abrasion resistance, allowing it to be used in applications where greater durability is required.

### Experimental device

**Figure 1:**
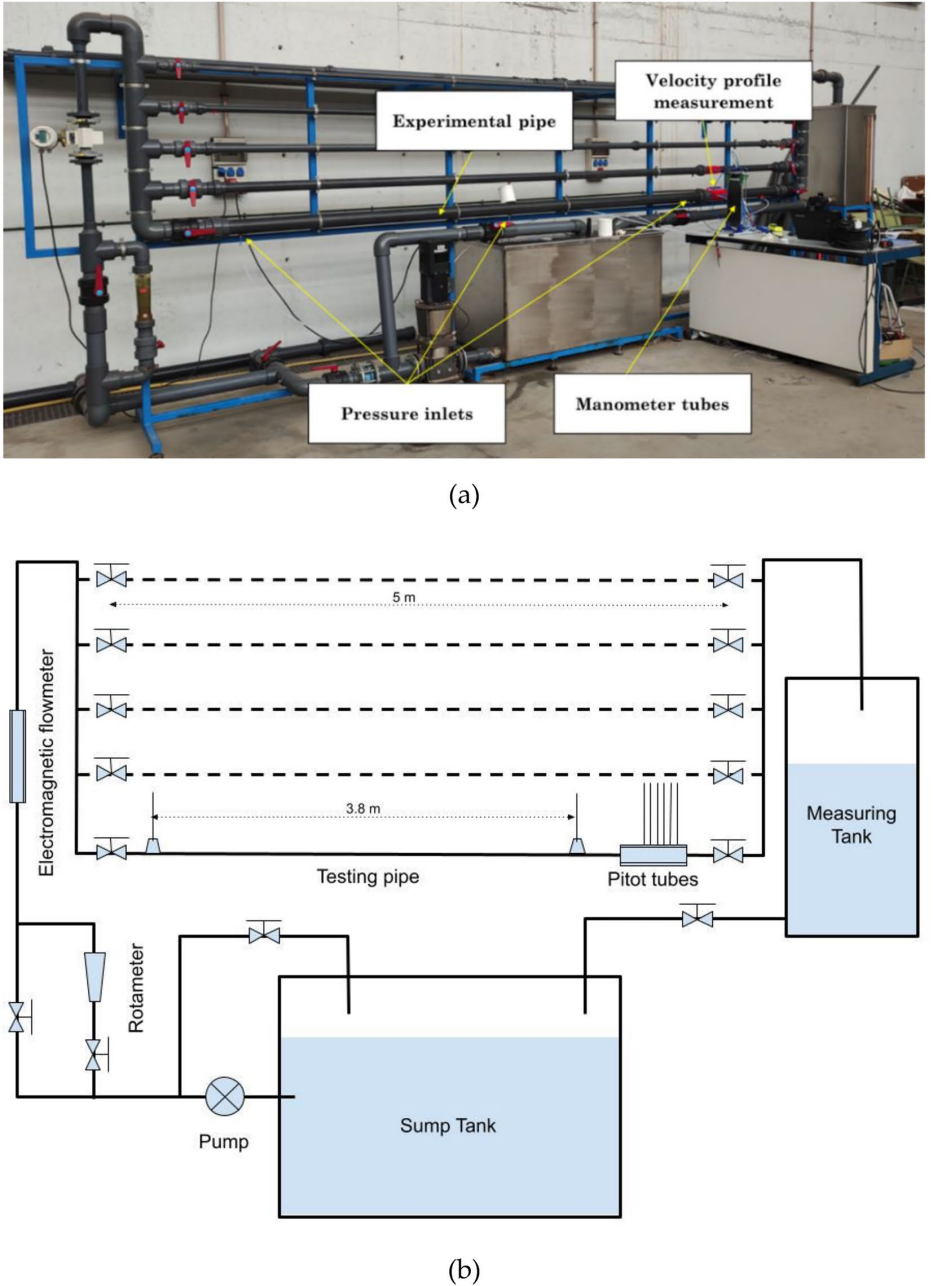
Picture (**a**) and scheme (**b**) of the pipe bench in the hydraulics laboratory of the University of Almería (Spain).

The project was carried out in the hydraulics laboratory of the University of Almería (Scientfic-Technical Building CITE II). This laboratory has a pipe bench specifically designed for testing both pipes and accessories (Fig. 1).

The bench has five lines of Polyvinylchloride (PVC) pipes of different diameters. These pipes are about five meters long and have valves on both sides of the line to open and close the flow of water. Water is drawn from a sump tank by a variable speed vertical pump. The device includes three procedures to measure flow. Two different kinds of flowmeters are installed, namely a rotameter and an ultrasonic flowmeter. The rotameter is a variable section flowmeter, whereas the ultrasonic flowmeter makes use of the Doppler effect to measure flow velocity. The measurement range of the rotameter spans from 1 to 10 m^3^/h with a precision of 0.2 m^3^/h. The measurement of the rotameter lies outside the flowrate used in this experiment, so this device was not used to measure flow. The ultrasonic flowmeter was the DE43F model of the FXE4000 (COPA-XE) series, manufactured by the company ABB and whose diameter is 40 mm. The velocity range of measurement is from 0.5 and 10 m/s, with a measurement error lower than 0.5%. In addition, there is a measuring tank that allows monitoring of flow using a gravimetric method. After passing through the measuring tank, water flows back to the sump tank. Three pressure taps were placed in each testing pipe. One was placed at each end of the pipe to measure the head losses. The separation between extreme pressure tubes was 3.8 m. The third pressure tube was located in the middle of the extreme tubes to validate their measurement.

Two different PVC pipes with different diameters were tested (PVC90 and PVC63). The working pressure of both pipes was 6 atm and the interior diameters were 84 and 59 mm, respectively. The estimated absolute roughness of the PVC pipe was 0.0015 mm, which results in a relative roughness of 1.79 10^–5^ and 2.54 10^–05^ for each diameter, respectively. Both uncoated and coated pipes were tested for every diameter size, which accounts for a total of 4 experimental pipes.

### Measurement of the velocity profile

Several procedures can be used to measure the velocity profile of fluid flow. According to Ayegba and Edomwonyi-Otu^29^, noninvasive techniques for measuring flow fields include point (e.g., laser Doppler velocimetry, LDV, laser Doppler anemometry, LDA), plane (e.g., particle image velocimetry, PIV) and volume (e.g., holographic particle image velocimetry, HPIV) and nuclear magnetic resonance velocimetry (NMRV) techniques. LDA/LDV relies on the laser property to create interference between intersecting beams in the flow under investigation. For LDA to measure a flow profile, the flow is seeded with small particles capable of following the fluid flow accurately, yet large enough for scattering the laser beams when the particles pass through the test section. Phase Doppler anemometry is an improvement on LDA which can measure the particle size of seeds. NMRV is a noninvasive technique that has been widely utilized in hospitals. When employing the NMRV technique, the media to be measured is positioned in a static magnetic field and a strong magnetic pulse is then applied to it. The recovery is measured from the disruption of the spin of the nuclei. If a single plane magnetic pulse is applied to flowing fluid media, it becomes possible to measure flow development incrementally over time, similar to particle tagging using dye tracers, followed by monitoring the resulting bands^29,30^.

The advantages of these image-based methods are that they are not invasive nor alter the flow field in any way, and they can be used to analyze specific regions of interest, describing the characteristics of the velocity field in said areas. However, the disadvantage is the need for costly and specialized equipment. In addition, most of these methods are not suitable for measuring the flow field in opaque PVC pipes.

**Figure 2:**
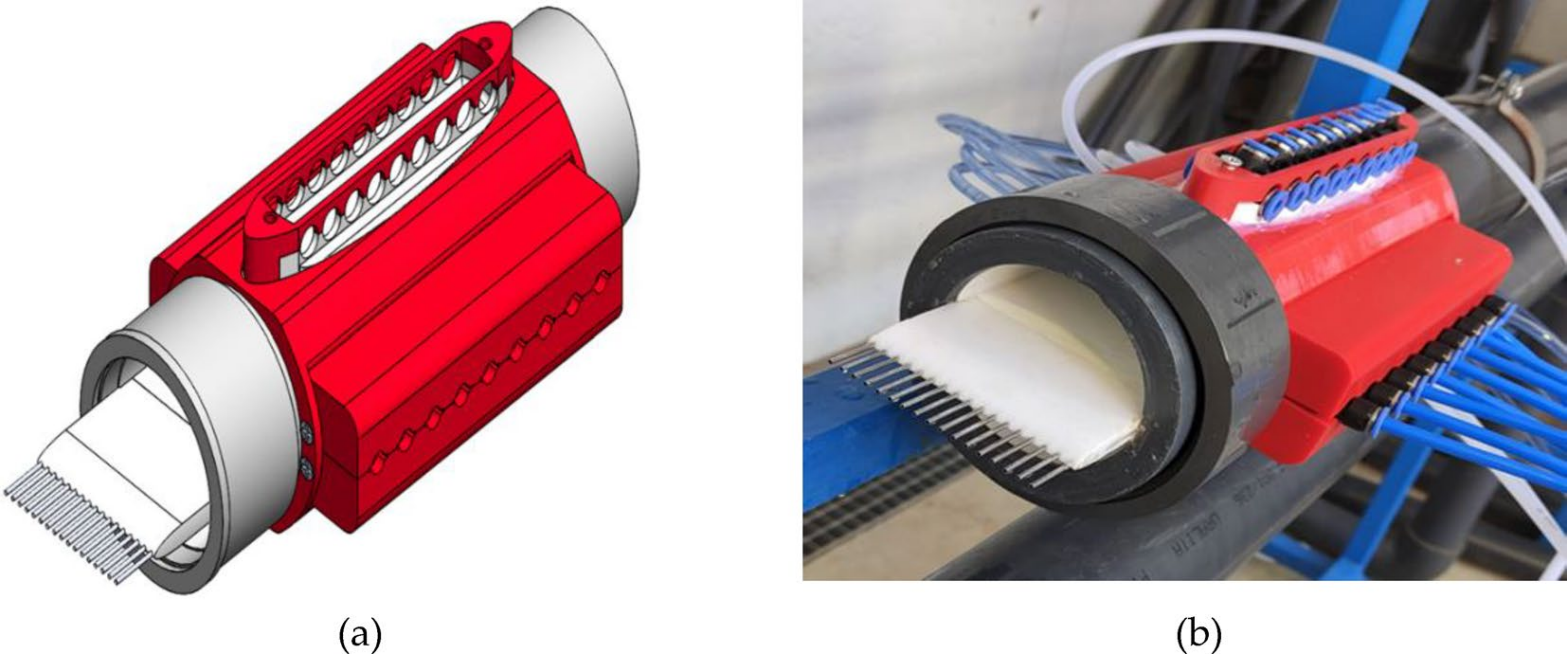
Device for the water velocity profile measurement. (**a**) 3D CAD drawing and (**b**) manufactured device installed in the hydraulics bench.

In this work, to measure the flow profile, a simpler yet precise method was used. We measured flow velocity in a defined set of streamlines at different positions within the axial plane of the pipe using a set of pitot tubes. For this purpose, a device was specifically designed and manufactured using Fused Deposition Modeling (FDM) techniques. The design of this accessory was carried out using the 3D Computer Aided Design software Solidworks. The designed device was then manufactured using a Creativity Ender 3 Pro 3D printer (Fig. 2).

Two devices were made: one for the 90 mm diameter PVC pipe, equipped with a total of 17 pitot tubes, and another for the 63 mm pipe, with 11 pitot tubes. The flow around these devices was simulated using Flow Simulation software to analyze their hydrodynamic performance and ensure minimal disturbance to the flow.

This pitot tube device was placed after the section of the pipe in which the head losses were measured as to not disturb the fluid flow.

**Figure 3:**
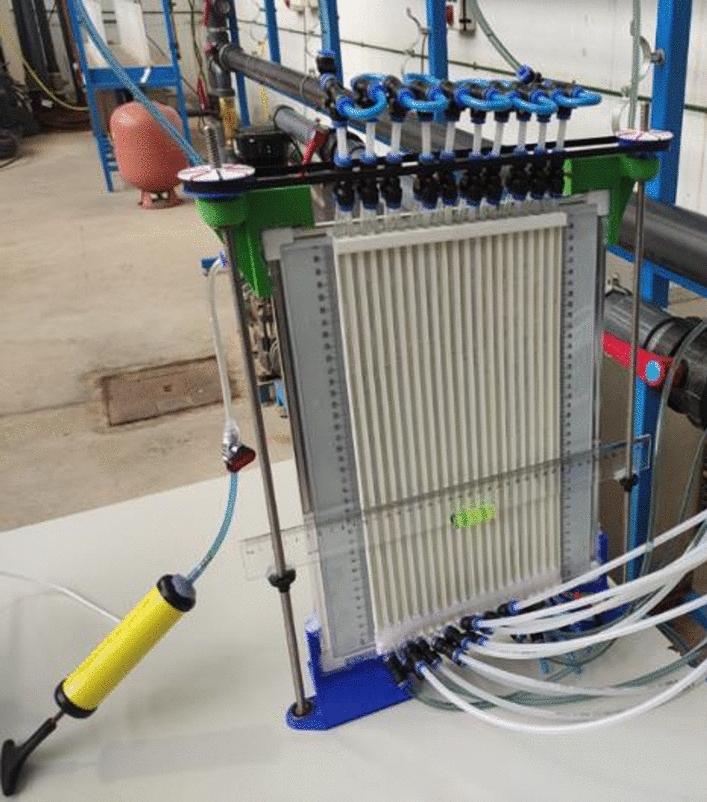
Bank of piezometric tubes to measure the pressure head differences.

All the pressure tubes were connected to a differential manometer composed of a set of manometric tubes enabling the measurement of pressure differences based on the water level differences in the tubes. This battery of manometric tubes was also specifically designed and manufactured for this work, involving all the previously described techniques (Fig. 3). This improved differential manometer had the following features: an increased number of 20 manometer tubes, quick fittings to easily connect the different measurement tubes to a valve to close the circuit and maintain pressure, 4 mm diameter tubes to reduce the size of the meniscus and the effect of capillarity. It also featured a mechanism with a horizontal ruler that made it easier to read the water levels, thus making them more accurate.

**Figure 4:**
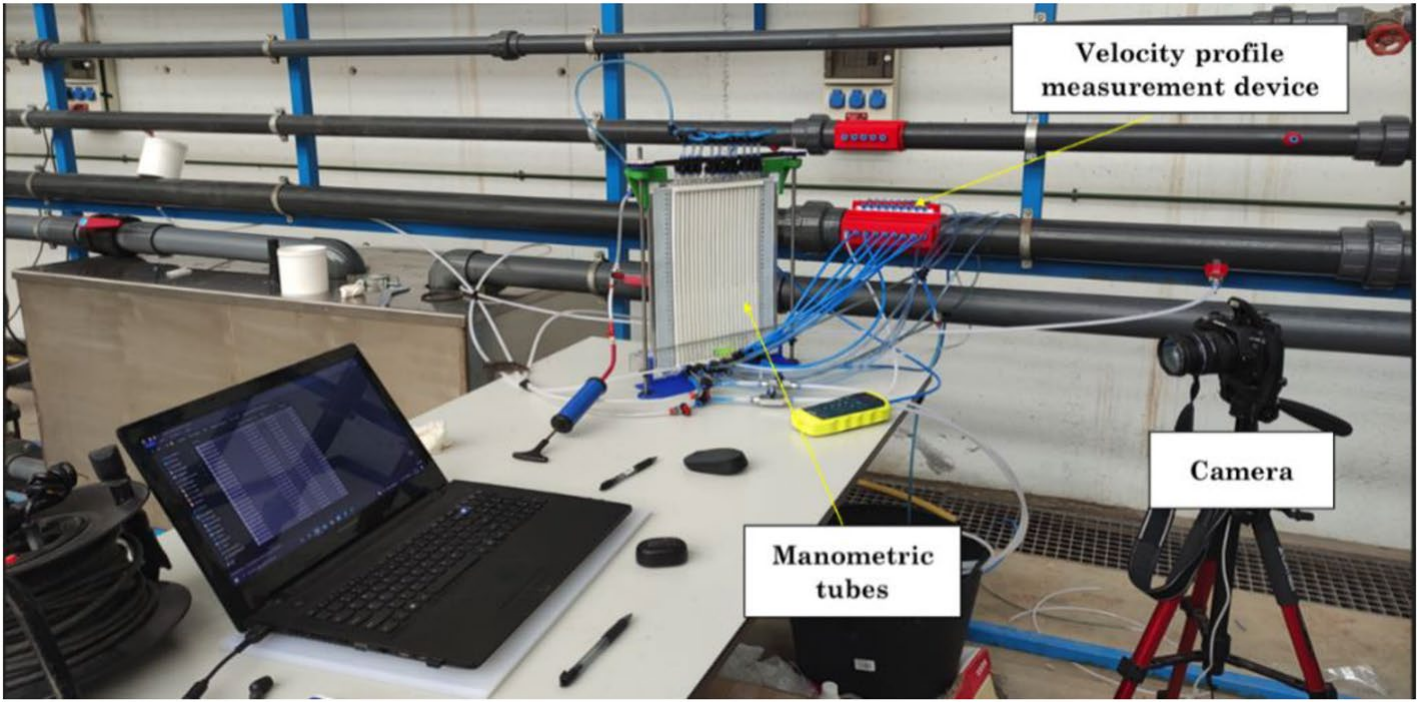
Measuring devices.

To maximize the accuracy of the measurements, a camera was used to take a snapshot of the set of pressure tubes to read the water levels simultaneously by processing the static image. Figure 4 shows a picture of the measurement device and methods used in this work.

### Hydrophobic coating application

**Figure 5:**
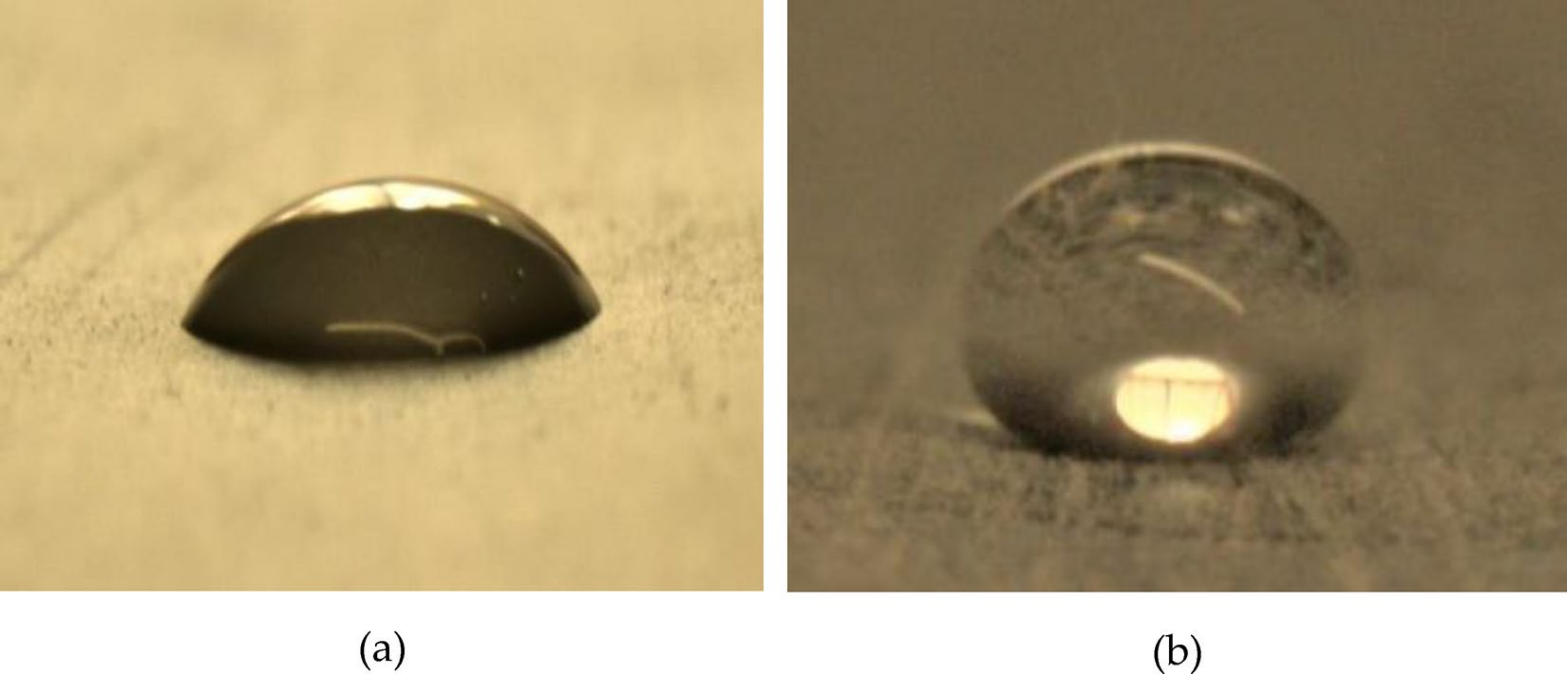
Photograph showing a drop of water poured into an uncoated pipe (**a**) and into a coated pipe showing its superhydrophobic properties (**b**).

One of the main challenges of this research was to apply a uniform coating of hydrophobic product to the inner wall of the pipe. The most efficient procedure was to apply the hydrophobic product nebulized in a pressurized air flow using a spray gun. The product was applied at one end of the pipe, which was kept closed so the mixture of air and product flowed towards the opposite end of the pipe while depositing the product on the interior wall. Several short nebulization operations were performed with controlled rotations of the pipe so the product could settle uniformly inside. After every nebulization, heat was applied with an air dryer so the product could dry before applying the next coating. After the application of the product was finished, the hydrophobic effect on the inner wall of the pipes was tested by analyzing the shape of drops of water poured into the pipe to ensure that the contact angle was compatible with the superhydrophobic properties of the product (Fig. 5).

### Experimental procedure

The experiment was performed using two PVC pipe diameters (90 and 63 mm), and both treated and untreated pipes were tested for these two diameters. For every pipe, several tests with different flowrates were carried out. The flowrate was regulated by varying the velocity of the pump and adjusting the opening of the valves. The flowrate was measured by the ultrasonic flowmeter and using the gravimetric method to validate the readings. For every test, the differential pressure at both ends of the pipe was read to calculate the head losses (also called drag reduction). Then, the flow velocity profile was registered by reading the water levels in the pressure tubes.

The head losses (*hf*), expressed as meters of water column, were computed as the difference of the pressure heads at the beginning and end of the pipe (Eq. 1):

1 $$hf=\frac{{p}_{o}-{p}_{1}}{\gamma }=\frac{\Delta p}{\gamma }$$

where: ${p}_{o}$ = pressure at the beginning of the pipe (in Pa), ${p}_{1}$= pressure at the end of the pipe (in Pa) and $\gamma$ = specific weight of the water (in N/m^3^).

One way to express the effect of the hydrophobic product on fluid flow is to calculate the friction coefficient (*f*). The friction coefficient is a non-dimensional parameter that depends on the Reynolds number and the relative roughness of the pipe. The friction factor values for every hydraulic regime are depicted in the Moody chart^31^. This variable can be obtained by solving for *f* in the Darcy-Weisbach equation (Eq. 2) from the experimental values of *hf* (Eq. 1) and the measured flowrate.

2 $$f=\frac{hf\cdot D\cdot 2g}{{U}^{2}\cdot L}$$

Another way to express the reduction in the energy consumption is the drag reduction percentage (%DR) along the pipeline. This parameter that has been extensively used in many research works dealing with the topic of drag reducing polymers (DRPs)^8,11,17^. This index can be defined in the context of this work as relative pressure difference (or head difference) between non-treated (subscript N) and treated pipes (subscript T).

3 $$\%DR=\frac{{\Delta p}_{N}-{\Delta p}_{T}}{{\Delta p}_{N}}\cdot 100$$

The experimental velocity profiles measured in treated and untreated pipes were compared and fitted to the most common empirical velocity profile models. In the central region of the flow, the outer turbulent layer, both power-law (Eq. 4) and logistics (Eq. 5) expressions have been proposed to accurately fit the flow velocity profile^32^.

4 $$\frac{u}{{u}_{max}}={\left(\frac{y}{{r}_{0}}\right)}^{1/n}$$

5 $$\frac{{u}_{max}-u}{{u}^{*}}=2.5 ln\left(\frac{{r}_{0}}{y}\right)$$

where: *u* = velocity of the streamline located at a distance *y* from the pipe wall, *u*_*max*_ = maximum velocity of flow profile that corresponds to the velocity in the centerline, *y* is the distance from the pipe wall, *r*_*o*_ is the radius of the pipe, *n* is a parameter that depends upon the flow regime and ${u}^{*}$ is the friction velocity, which can be expressed as:

6 $${u}^{*}=\sqrt{\frac{{\tau }_{w}}{\rho }}$$

where: ${\tau }_{w}$ is the shear stress at the contour of the pipe and $\rho$ the density of the fluid.

The statistical analysis of the experimental data was performed with the Ms-Excel^®^ and the Statgraphics^®^ software packages.

## Results and discussion

### Measuring the energy reduction

#### Testing the uncoated pipes and calibrating the measuring device

First, the two uncoated PVC pipes were tested with the aim of calibrating the experimental device and assessing its accuracy and measuring errors. The results of the measurement for both diameter pipes are shown in Table 1.

**Table 1:** Testing results of the untreated pipes.

Test	PVC90				PVC63			
	Flow (l/s)	Re	hf (mm)	f	Flow (l/s)	Re	hf (mm)	f
1	1.53	2.31E+04	4.5	0.0257	0.47	1.02E+04	3.0	0.0305
2	1.97	2.98E+04	7.0	0.0240	0.83	1.79E+04	8.0	0.0261
3	2.44	3.69E+04	10.0	0.0223	1.17	2.51E+04	14.5	0.0241
4	2.72	4.11E+04	12.0	0.0216	1.56	3.35E+04	24.5	0.0229
5	3.06	4.62E+04	15.0	0.0215	1.83	3.94E+04	32.5	0.0219
6	3.42	5.16E+04	18.0	0.0206	2.00	4.30E+04	38.0	0.0215
7	3.71	5.60E+04	21.0	0.0204	2.39	5.14E+04	52.0	0.0206
8	4.03	6.09E+04	24.5	0.0202	2.58	5.56E+04	60.0	0.0204
9	4.69	7.09E+04	32.0	0.0194	2.89	6.22E+04	73.5	0.0199
10	5.22	7.89E+04	38.5	0.0189	3.17	6.81E+04	86.5	0.0195
11					3.44	7.41E+04	100.5	0.0192
12					3.81	8.19E+04	120.0	0.0188

**Figure 6:**
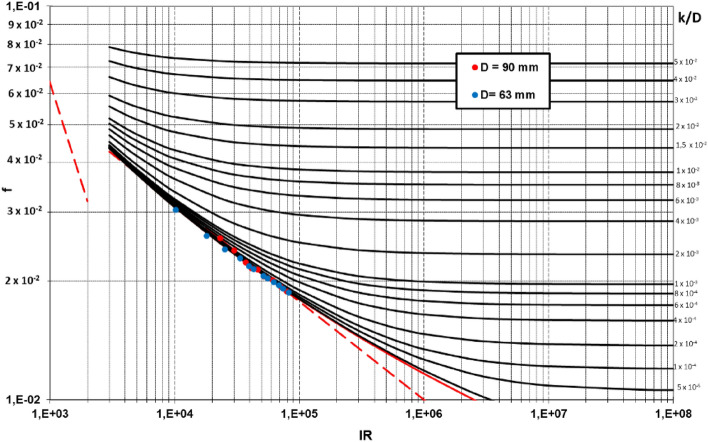
Moody charts depicting the experimental friction factors values for the untreated PVC pipes.

The calculated friction factor values are depicted in the Moody chart. These values lie on the hydraulic smooth curve, as was expected for both pipes (see Fig. 6). The experimental absolute roughness value *k* was derived by fitting this parameter from the experimental points to the theoretical Darcy-Weisbach equation by minimizing the square errors between the experimental *f* values and the fitted ones. The resulting experimental *k* value was 0.002 mm, which is close to that recommended by the manufacturer. Using this adjusted *k* value, the coefficient of determination (R^2^) between the theoretical and experimental *f* curves were higher than 0.99 for both pipes. The relative error was 1.05% and 0.93%. These data proved that the experimental data fitted well to the theoretical curve.

**Figure 7:**
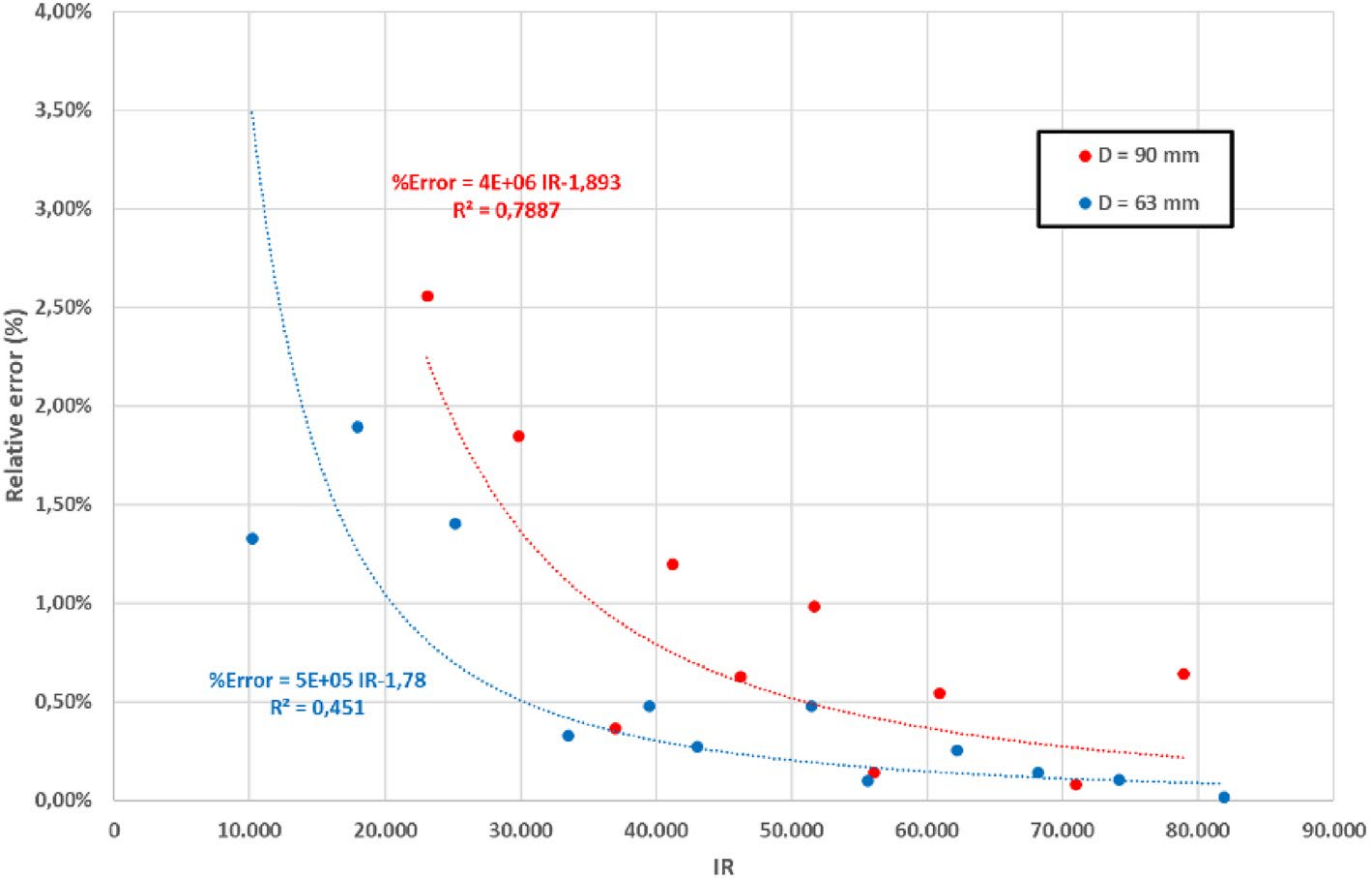
Absolute errors of the measurement of the Head Losses for the untreated PVC pipes.

To estimate the relative errors in the measurement of the head losses (pressure head differences), empirical values were compared with the theoretical ones obtained for the theoretical curve. These errors were always lower than 5% for both pipes and for any Reynolds number (Re) value. The accuracy of the measurement was lower for low Re values, as depicted in Fig. 7, because the measured pressure head differences (expressed as mm of water column) were smaller, and the accuracy of the pressure tubes was 1 mm. These results confirmed the accuracy of the head loss measurement.

The mean Absolute Error (MAE) and Root mean squared error (RMSE) of the observations compared to the theoretical head loss are 0.11 and 0.13 mm, respectively, for the 90 mm pipe and 0.38 and 0.42 mm for the 63 mm pipe. These errors expressed in relative terms are 0.6% and 0.7% for the 90 mm pipe and 0.36% and 0.40% for the 63 mm pipe. These figures confirm the accuracy of the measurements. Nevertheless, the accuracy of the measurement considerably improved with rising Reynolds values, primarily due to the precision of the measuring device. The use of differential pressure tubes with a resolution of 1 mm of water column resulted in increased measurement errors for lower head loss values. This trend is clearly shown in Fig. 7. On the other hand, no systematic sources of errors were observed in the measurement procedure.

#### Head loss reduction for the coated pipes

The head losses of the pipes with the hydrophobic coating were then tested and the head losses and friction coefficients were computed from the experimental data. Table 2 shows the values found in the experiment.

**Table 2:** Testing results of the treated pipes.

Test	PVC90				PVC63			
	Flow (l/s)	Re	hf (mm)	f	Flow (l/s)	Re	hf (mm)	f
1	0.88	1.33E+04	1.0	0.0171	1.0	7.17E+03	1.0	0.0204
2	1.31	1.97E+04	2.5	0.0196	4.0	1.37E+04	4.0	0.0222
3	1.50	2.27E+04	3.5	0.0208	8.5	1.91E+04	8.5	0.0244
4	1.75	2.64E+04	5.0	0.0218	14.5	2.51E+04	14.5	0.0241
5	2.08	3.15E+04	7.5	0.0231	25.0	3.41E+04	25.0	0.0226
6	2.39	3.61E+04	10.0	0.0234	34.5	4.06E+04	34.5	0.0219
7	2.75	4.16E+04	12.0	0.0212	44.0	4.72E+04	44.0	0.0207
8	2.97	4.49E+04	14.0	0.0212	51.5	5.20E+04	51.5	0.0200
9	3.25	4.91E+04	17.0	0.0215	60.0	5.56E+04	60.0	0.0204
10	3.92	5.92E+04	23.0	0.0200	75.0	6.34E+04	75.0	0.0196
	4.50	6.80E+04	29.5	0.0195	118.5	8.19E+04	118.5	0.0185
	5.11	7.72E+04	37.0	0.0189	130.0	8.85E+04	130.0	0.0174

**Figure 8:**
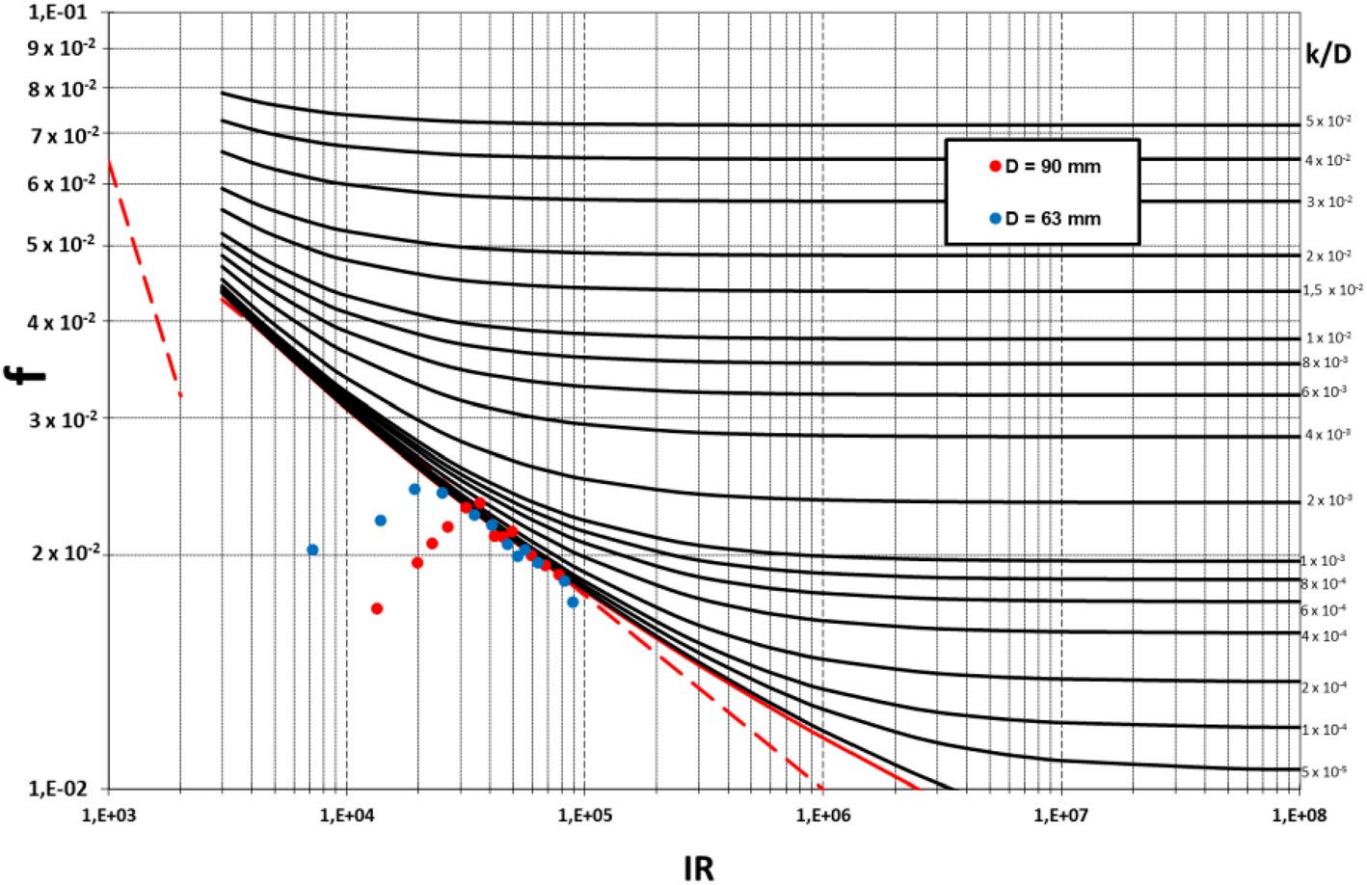
Moody charts depicting the experimental friction factors values for the treated PVC pipes.

Figure 8 depicts the *f* values obtained for the coated pipes. These results show that the hydrophobic coating did influence the head losses for relatively small Re values. As shown in the graph, the experimental friction factor curves for the treated pipes display an abnormal shape, since their values were considerably lower than those expected for a hydraulically smooth pipe. The values of these friction factors rise as the Reynolds number values increase, tending to progressively follow the hydraulically smooth curve. This behavior was observed for both pipes, although the pipe with a smaller diameter size (higher relative roughness) reached this hydraulically smooth curve for lower Reynolds number values than the pipe with a larger diameter (lower relative roughness). This fact may indicate that for a transition regime with moderate turbulence the hydrophobic product induces a partial slip condition in the interface between the fluid and the contour of the pipe. The effect of this alleged slip condition tends to vanish for a regime with a higher Reynolds number and stronger turbulence. This occurrence could be explained by noting that the laminar sublayer reduces its size for a higher Reynolds number, thus interfering with this slip condition. This could also explain the varied performance of pipes with different relative roughness. As can be seen in the graph, the pipe with higher relative roughness (the smaller diameter, PVC63) tended to the hydraulically smooth curve for lower Re numbers compared to the pipe with a lower relative roughness (PVC90). This is consistent with the theory that the interaction between roughness and the turbulent layer interferes with the slip condition between the fluid and the pipe wall.

**Figure 9:**
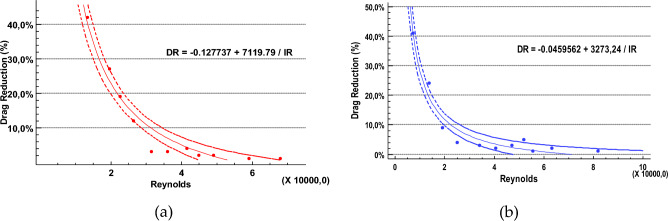
Experimental Drag Reduction and adjusted equations as a function of the Reynolds Number, (**a**) For the 90 mm pipe and (**b**) for the 63 mm pipe.

The particular effect of energy loss reduction becomes even clearer if the percentage of drag reduction (%DR) is considered. Figure 9 shows the calculated %DR for both pipes. The %DR grows significantly for relatively low Reynolds values, whereas it tends asymptotically to zero for increasing values of the Reynolds number. This trend is again repeated for both pipes but for slightly different values of the Reynolds number. These results extend those obtained by Watanabe et al.^27^, who reported a 14% drag reduction for a laminar flow to a transition regime. Figure 9 shows the experimental %DR based on the Reynolds number and the fitted models. This finding could hold significant implications for practical applications, especially in fluid systems in which operating conditions frequently fall within lower Reynolds number ranges. This is the case of relatively viscous fluids, such as oil. In other less viscous fluids, such as water, energy savings are expected to be considerably lower due to typically higher Re numbers in water systems.

Reciprocal-Re models fitted very well the experimental results for both pipes, with a Correlation Coefficient of 0.98 and an adjusted R^2^ value of 0.94. For the PVC90 pipe, values of 3.32% and 2.39% were obtained for the Standard Error of Estimates and Mean absolute error, respectively. Similarly, for the PVC63 pipe, the values were a Correlation Coefficient of 0.97, an adjusted R^2^ value of 0.95, with 2.92% for the Standard Error of Estimates and 2.14% for the Mean absolute error.

Although these simple regression models fitted the experimental data very well, an exploration for a non-linear multiple regression model to include the effect of the relative roughness of the pipe. The model in Eq. (7) was found to fit the experimental data of both pipes satisfactorily well, considering both Re and *k*/*D* variables. It yielded an adjusted R^2^ value of 0.90, with values of 4.08% for the Standard Error of Estimates and 2.96% for the Mean absolute error:

7 $$DR = \frac{{ - 1.45\;\;10^{ - 6 + } + \frac{{9.69\;\;10^{ - 2} }}{IR}}}{\frac{k}{D}}$$

### Effect of the hydrophobic coating on the velocity profile

In this section, the flow profiles for both treated and untreated pipes are compared to determine whether the measured head loss differences can be explained by changes in the velocity profile of the fluid.

#### Velocity profiles of the untreated pipes

The velocity profiles were measured in both uncoated and coated pipes for different Re values. These velocity profiles were fitted to the Power-Law and Logistic models described in "Experimental procedure" section.

**Figure 10:**
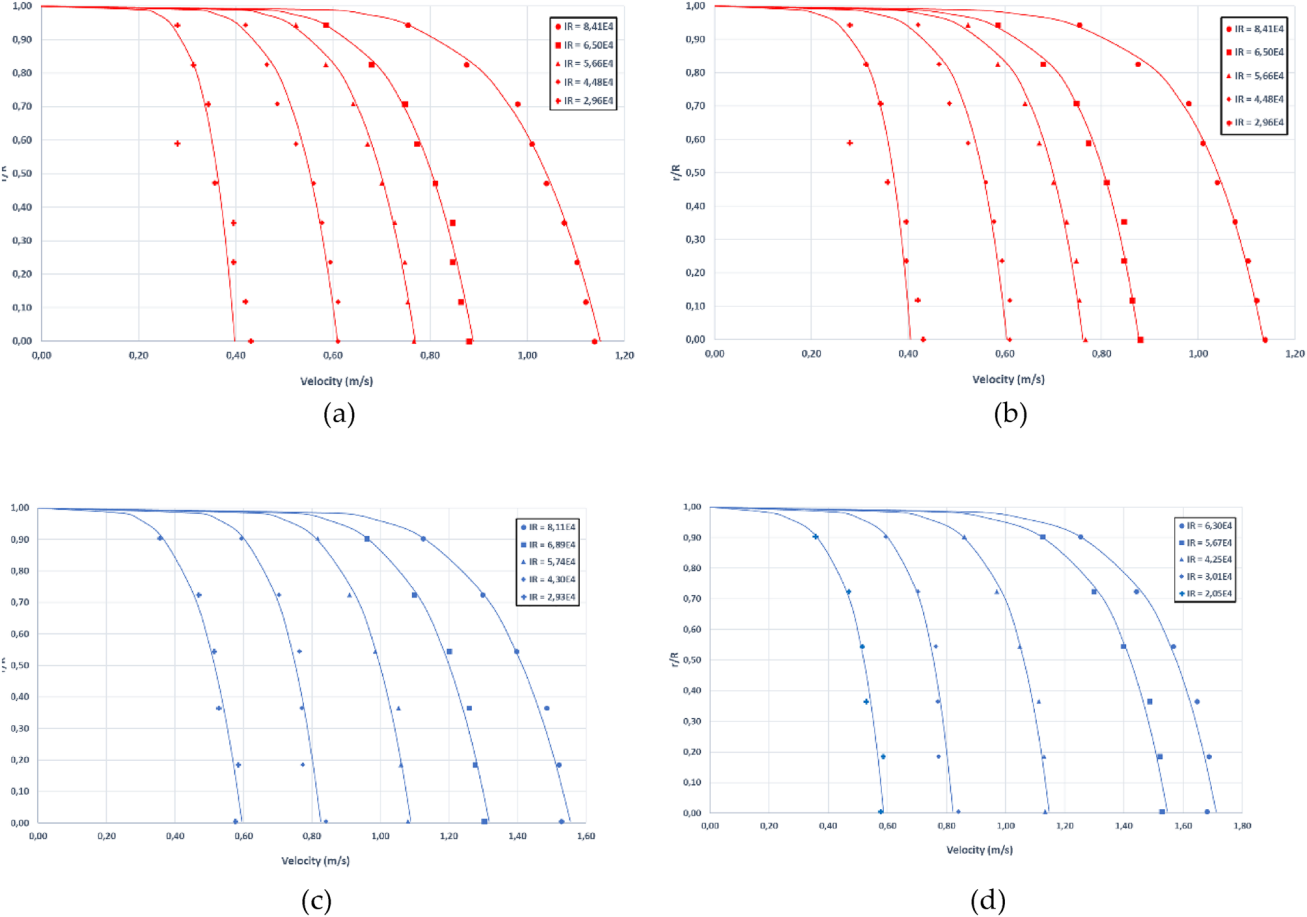
Flow velocity profiles for the PVC90 pipe according to the Power-law (**a**) and Logistic model (**b**) and flow velocity profiles for the PVC63 pipe according to the Power-law (**c**) and Logistic model (**d**).

Figure 10 shows the experimental data and fitted velocity profiles according to the Power-Law model for PVC90 (a) and PVC63 (b) and according to the Logistic model for PVC90 (c) and PVC63 (d), respectively, for 5 selected Re values.

Table 3 shows the adjusted parameters of the Power-Law fitting for the two diameters of the untreated pipes to validate and verify the accuracy of the measurement device.

**Table 3:** Fitted parameters and goodness of fit indicators for the Power-law velocity profile model for both PVC90 and PVC63 untreated pipes.

	Test	Re	V_med_	V_max_	n	R^2^	MAE		RMSE	
							(m/s)	%	(m/s)	%
PVC90	1	2.54E+04	0.30	0.34	7.0	0.64	0.0224	7.58	0.0274	9.03
	2	2.96E+04	0.35	0.40	7.3	0.63	0.0217	6.24	0.0299	8.44
	3	3.85E+04	0.46	0.55	5.2	0.99	0.0049	1.11	0.0062	1.36
	4	4.48E+04	0.53	0.61	7.1	0.95	0.0104	2.10	0.0128	2.39
	5	4.93E+04	0.59	0.68	6.3	0.98	0.0080	1.43	0.0097	1.64
	6	5.66E+04	0.68	0.77	7.2	0.99	0.0063	1.01	0.0078	1.15
	7	5.96E+04	0.71	0.82	6.2	1.00	0.0044	0.65	0.0055	0.77
	8	6.50E+04	0.78	0.89	6.9	0.99	0.0069	0.96	0.0081	1.04
	9	7.58E+04	0.90	1.03	7.0	1.00	0.0052	0.60	0.0066	0.73
	10	8.41E+04	1.00	1.15	6.8	0.99	0.0088	0.94	0.0108	1.07
PVC63	1	9.03E+03	0.17	0.34	1.7	0.90	0.0329	15.01	0.0365	21.58
	2	1.44E+04	0.28	0.45	3.1	0.93	0.0223	6.67	0.0246	8.91
	3	2.05E+04	0.50	0.60	4.8	0.98	0.0130	2.76	0.0134	2.70
	4	2.62E+04	0.64	0.73	6.8	0.99	0.0052	0.89	0.0065	1.03
	5	3.01E+04	0.73	0.83	7.4	0.96	0.0150	2.20	0.0172	2.35
	6	3.31E+04	0.81	0.90	7.8	0.98	0.0110	1.48	0.0124	1.53
	7	4.01E+04	0.98	1.09	7.9	0.98	0.0095	1.07	0.0124	1.27
	8	4.25E+04	1.03	1.15	7.9	0.99	0.0102	1.09	0.0130	1.26
	9	4.82E+04	1.17	1.32	7.4	0.99	0.0111	1.03	0.0124	1.06
	10	5.27E+04	1.28	1.43	8.0	0.99	0.0113	0.97	0.0140	1.09
	11	5.67E+04	1.38	1.56	7.3	0.99	0.0112	0.89	0.0153	1.11
	12	6.30E+04	1.53	1.72	7.4	0.99	0.0177	1.27	0.0215	1.40

The shape of the power law velocity profile depends on parameter *n* (see Eq. 4), which in turn depends on the hydraulic regime of the flow. The value of this parameter grows with rising Reynolds number values, and higher *n* values indicate that the flow profile is flatter since *n* in the power-law model is in the denominator of the exponent in Eq. (4), as expected for flows with a higher degree of turbulence. Regarding the maximum velocity ${u}_{max}$, although it was experimentally measured with a pitot tube located in the centerline, to minimize the errors produced by a single measurement, it was herein considered as another estimated parameter of the power-law model, along with parameter *n*. In this way, the velocity of the centerline is obtained not only from a single reading but also from the complete flow profile.

**Figure 11:**
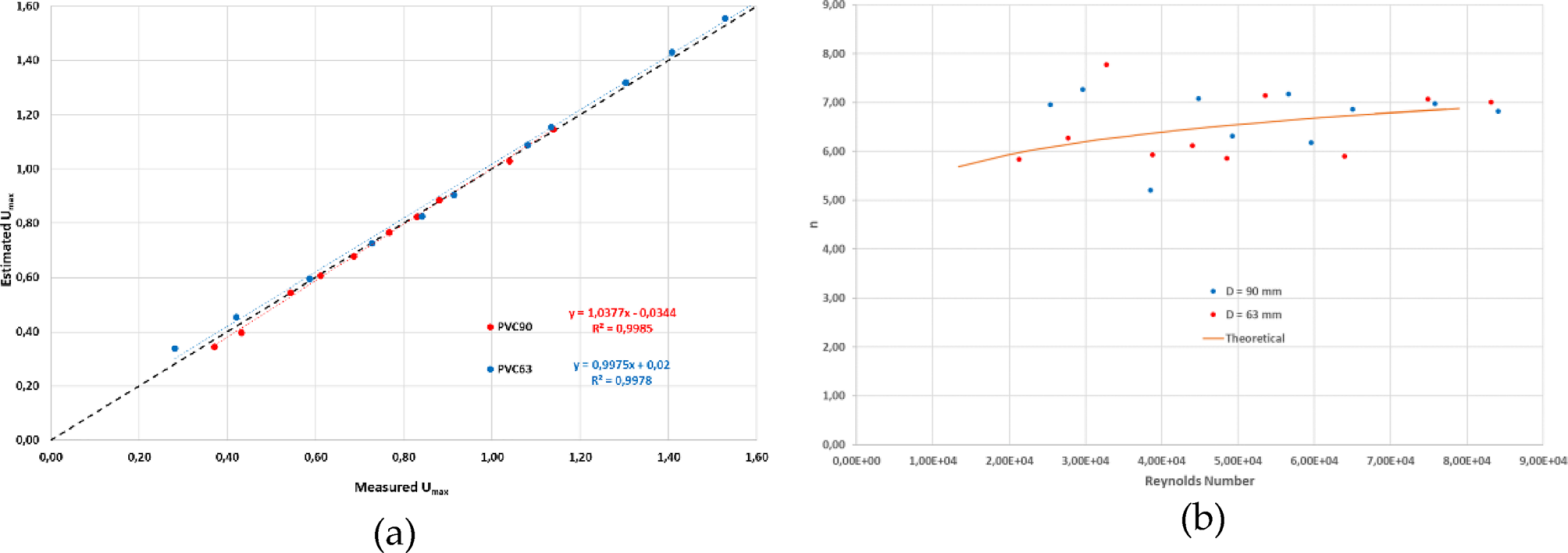
Comparison between measured *U*_*max*_ in both pipes and the fitted *U*_*max*_ for the Power-law model (**a**) and comparison between fitted and theoretical *n* values as a function of the Reynolds number for both pipes (**b**).

Figure 11a, shows measured versus adjusted ${u}_{max}$ values for both pipes. The relative errors decreased considerably for increasing Re numbers. The average relative MAE and RMSE for Re numbers higher than 3 10^4^ were 1.10 and 1.27% for the PVC90 pipe and 1.28 and 1.38% for the PVC63 pipe, respectively.

The adjusted values of *n* are compatible with a turbulent flow. Figure 11b depicts the adjusted *n* values of the power-law model as a function of the Reynolds number and the theoretical *n* values (continuous line) according to the relationships reported in Munson et al.^32^. In conclusion, very good agreement was found between measured velocity profiles and the Power-Law model.

To assess the accuracy of the flow profile measurement device for each pitot tube, the experimental velocity readings were compared with the fitted Power-Law velocities for every flow test in both pipes. The results are shown in Table 4.

**Table 4:** Fitted parameters and goodness of fit indicators for the Power-law velocity profile model for both PVC90 and PVC63 untreated pipes.

	Pitot tube	MAE		RMSE	
		(m/s)	%	(m/s)	%
PVC90	1	0.007	1.39	0.008	1.06
	2	0.009	1.71	0.018	2.19
	3	0.013	2.11	0.015	1.60
	4	0.008	1.23	0.018	1.92
	5	0.005	0.83	0.014	1.53
	6	0.007	1.08	0.027	2.72
	7	0.006	0.91	0.008	1.06
	8	0.012	1.63	0.018	2.19
	9	0.010	1.39	0.015	1.60
PVC63	1	0.006	0.85	0.008	1.74
	2	0.015	1.75	0.011	2.01
	3	0.012	1.30	0.015	2.51
	4	0.017	1.74	0.009	1.50
	5	0.011	1.21	0.007	1.14
	6	0.022	2.26	0.010	1.41

The accuracy of the measurements was satisfactory, with errors lower than 3% in all pitot tubes. In addition, no systematic errors were detected in any specific tube since they were all randomly distributed. These results validate the experimental device used in this work.

Table 5 shows the adjusted parameters for the logistic fitting along with the indicators of the goodness of fit for both pipes.

**Table5:** Fitted parameters and goodness of fit indicators for the Logistic velocity profile model for both PVC90 and PVC63 untreated pipes.

	Test	Re	K	u*	V_max_	R^2^	MAE		RMSE	
							(m/s)	%	(m/s)	%
PVC90	1	2.54E+04	− 0.04	0.0	0.35	0.63	0.0322	10.63	0.0034	12.06
	2	2.96E+04	− 0.05	0.0	0.40	0.68	0.0354	10.01	0.0048	11.68
	3	3.85E+04	− 0.08	0.0	0.54	0.99	0.0336	7.31	0.0061	9.46
	4	4.48E+04	− 0.07	0.0	0.60	0.94	0.0302	5.64	0.0076	7.48
	5	4.93E+04	− 0.09	0.0	0.67	0.97	0.0366	6.23	0.0099	8.04
	6	5.66E+04	− 0.09	0.0	0.76	0.98	0.0371	5.49	0.0093	6.90
	7	5.96E+04	− 0.11	0.0	0.82	0.99	0.0448	6.29	0.0125	7.94
	8	6.50E+04	− 0.11	0.0	0.88	0.99	0.0437	5.63	0.0105	7.11
	9	7.58E+04	− 0.12	0.0	1.02	0.99	0.0507	5.61	0.0145	7.26
	10	8.41E+04	− 0.14	0.1	1.13	0.99	0.0567	5.65	0.0168	7.11
PVC63	1	9.03E+03	− 0.09	0.04	0.30	0.96	0.0148	9.61	0.0002	10.31
	2	1.44E+04	− 0.10	0.04	0.44	0.97	0.0122	5.00	0.0002	5.79
	3	2.05E+04	− 0.10	0.04	0.59	0.98	0.0094	2.71	0.0000	3.14
	4	2.62E+04	− 0.09	0.04	0.72	0.99	0.0042	0.94	0.0000	1.20
	5	3.01E+04	− 0.10	0.04	0.82	0.95	0.0137	2.69	0.0000	3.19
	6	3.31E+04	− 0.10	0.04	0.90	0.98	0.0098	1.73	0.0000	2.05
	7	4.01E+04	− 0.12	0.05	1.08	0.98	0.0105	1.55	0.0001	1.97
	8	4.25E+04	− 0.13	0.05	1.15	0.98	0.0107	1.49	0.0000	1.75
	9	4.82E+04	− 0.15	0.06	1.31	0.99	0.0097	1.18	0.0000	1.36
	10	5.27E+04	− 0.15	0.06	1.42	0.99	0.0104	1.16	0.0000	1.38
	11	5.67E+04	− 0.18	0.07	1.55	0.99	0.0188	1.95	0.0000	1.44
	12	6.30E+04	− 0.20	0.08	1.71	0.99	0.0237	2.21	0.0000	1.73

These data indicate that the goodness of fit of the experimental data to the proposed logistic model was also very good, with R^2^ values close to unity, except for the measurement performed for low Reynolds numbers. The adjusted ${u}_{max}$ values using this model agreed well with both the measured values and with the power-law model. As expected, the u* values increased as the Reynolds numbers became higher. The measured flow profiles were compatible with the expected velocity profiles for an increasing turbulent flow regime.

Although both models fitted the experimental measurements quite well, the power-law model performed slightly better than the logistic one, especially for higher Re numbers. For lower Re numbers, it is not so clear which model is better due to the higher uncertainty and lower accuracy of the measurement.

#### Effect of the hydrophobic product on the velocity profile

**Figure 12:**
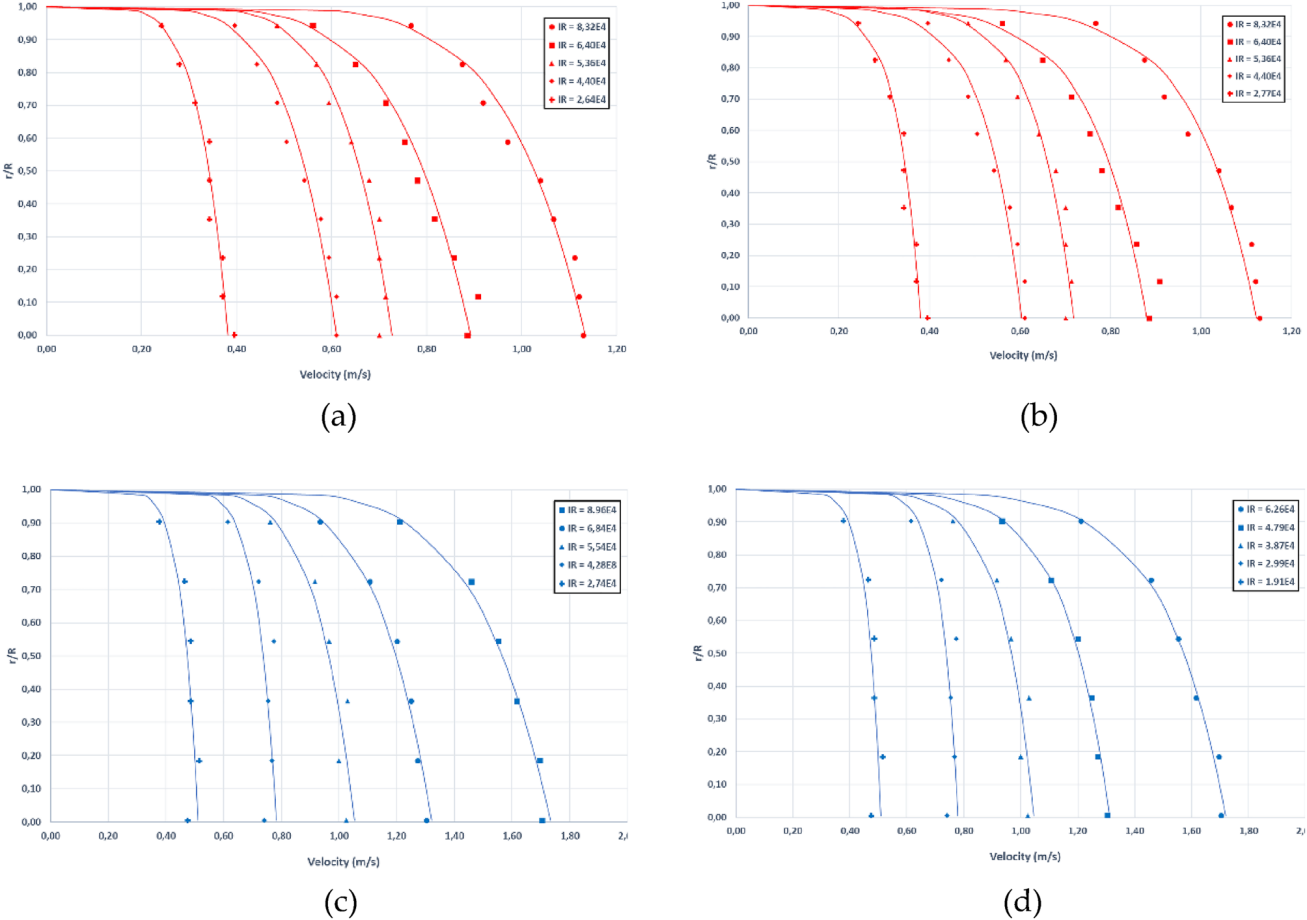
Flow velocity profiles for the coated pipes. PVC90 pipe according to the Power-law (**a**) and Logistic model (**b**) and flow velocity profiles for the PVC63 pipe according to the Power-law (**c**) and Logistic model (**d**).

With the aim of analyzing the effect of the hydrophobic coating in the velocity distribution, the velocity profiles measured in the treated pipes were also adjusted to both Power-Law and Logistic models. The velocity profiles are depicted in Fig. 12.

The fitting results for the Power-Law model are shown in Table 6, while Table 7 displays the results for the Logistic model.

**Table 6:** Fitted parameters and goodness of fit indicators for the Power-law velocity profile model for both PVC90 and PVC63 treated pipes.

	Test	Re	V_max_	n	R^2^	MAE		RMSE	
						(m/s)	%	(m/s)	%
PVC90	1	1.27E+04	0.19	3.6	0.85	0.0115	7.00	0.0136	8.95
	2	1.95E+04	0.28	4.5	0.89	0.0114	4.96	0.0141	6.07
	3	2.13E+04	0.30	5.8	0.84	0.0136	5.35	0.0159	6.27
	4	2.77E+04	0.38	6.3	0.98	0.0066	2.06	0.0084	2.55
	5	3.28E+04	0.44	7.8	0.94	0.0089	2.48	0.0107	2.73
	6	3.88E+04	0.54	5.9	0.96	0.0094	2.15	0.0117	2.54
	7	4.40E+04	0.61	6.1	0.97	0.0118	2.37	0.0132	2.52
	8	4.86E+04	0.68	5.9	0.99	0.0099	1.85	0.0120	2.06
	9	5.36E+04	0.72	7.1	0.98	0.0087	1.53	0.0130	2.03
	10	6.40E+04	0.89	5.9	0.98	0.0143	1.97	0.0165	2.16
	11	7.48E+04	1.01	7.1	0.99	0.0098	1.24	0.0148	1.66
	12	8.32E+04	1.13	7.0	0.98	0.0135	1.46	0.0172	1.73
PVC63	1	1.11E+04	0.27	2.2	0.78	0.0304	12.40	0.0368	19.49
	2	1.41E+04	0.31	3.3	0.96	0.0102	3.91	0.0120	5.03
	3	2.13E+04	0.41	6.4	0.95	0.0095	2.88	0.0105	2.91
	4	2.74E+04	0.51	8.9	0.80	0.0178	4.28	0.0206	4.43
	5	3.68E+04	0.69	8.2	0.83	0.0241	4.33	0.0272	4.35
	6	4.28E+04	0.78	11.4	0.77	0.0221	3.52	0.0276	3.79
	7	4.93E+04	0.92	9.4	0.89	0.0222	3.03	0.0253	3.02
	8	5.54E+04	1.05	7.8	0.94	0.0242	2.86	0.0252	2.67
	9	5.99E+04	1.15	7.2	0.97	0.0198	2.14	0.0205	2.01
	10	6.86E+04	1.32	7.0	0.99	0.0132	1.22	0.0139	1.19
	11	8.96E+04	1.73	6.7	0.99	0.0162	1.15	0.0181	1.19
	12	9.36E+04	1.80	7.0	0.98	0.0206	1.42	0.0261	1.64

**Table 7:** Fitted parameters and goodness of fit indicators for the Logistic velocity profile model for both PVC90 and PVC63 treated pipes.

	Test	Re	K	u*	V_max_	R^2^	MAE		RMSE	
							(m/s)	%	(m/s)	%
PVC90	1	1.27E+04	− 0.04	0.0	0.19	0.01	0.0140	9.26	0.0005	10.77
	2	1.95E+04	− 0.04	0.0	0.28	0.01	0.0101	4.34	0.0001	5.27
	3	2.13E+04	− 0.04	0.0	0.30	0.01	0.0152	6.00	0.0006	7.07
	4	2.77E+04	− 0.05	0.0	0.38	0.00	0.0083	2.52	0.0001	2.86
	5	3.28E+04	− 0.05	0.0	0.44	0.01	0.0104	2.67	0.0002	3.08
	6	3.88E+04	− 0.07	0.0	0.53	0.01	0.0131	2.84	0.0005	3.36
	7	4.40E+04	− 0.08	0.0	0.60	0.01	0.0160	3.05	0.0007	3.30
	8	4.86E+04	− 0.09	0.0	0.66	0.00	0.0088	1.51	0.0001	1.92
	9	5.36E+04	− 0.08	0.0	0.72	0.01	0.0104	1.63	0.0000	1.97
	10	6.40E+04	− 0.12	0.0	0.88	0.01	0.0192	2.52	0.0008	2.87
	11	7.48E+04	− 0.12	0.0	1.00	0.01	0.0097	1.08	0.0000	1.45
	12	8.32E+04	− 0.13	0.1	1.12	0.01	0.0185	1.87	0.0009	2.26
PVC63	1	1.11E+04	− 0.07	0.03	0.26	0.70	0.0318	16.83	0.0000	20.35
	2	1.41E+04	− 0.07	0.03	0.31	0.92	0.0132	5.53	0.0002	6.65
	3	2.13E+04	− 0.05	0.02	0.41	0.96	0.0081	2.22	0.0000	2.40
	4	2.74E+04	− 0.05	0.02	0.51	0.80	0.0169	3.64	0.0003	4.16
	5	3.68E+04	− 0.07	0.03	0.69	0.83	0.0226	3.60	0.0005	4.00
	6	4.28E+04	− 0.06	0.02	0.78	0.76	0.0208	2.86	0.0006	3.60
	7	4.93E+04	− 0.08	0.03	0.91	0.89	0.0200	2.38	0.0003	2.78
	8	5.54E+04	− 0.11	0.05	1.05	0.94	0.0204	2.17	0.0003	2.32
	9	5.99E+04	− 0.13	0.05	1.14	0.98	0.0151	1.48	0.0002	1.57
	10	6.86E+04	− 0.16	0.06	1.31	1.00	0.0074	0.64	0.0000	0.73
	11	8.96E+04	− 0.22	0.09	1.72	0.99	0.0160	1.05	0.0001	0.79
	12	9.36E+04	− 0.22	0.09	1.79	0.98	0.0250	1.57	0.0000	1.33

**Figure 13:**
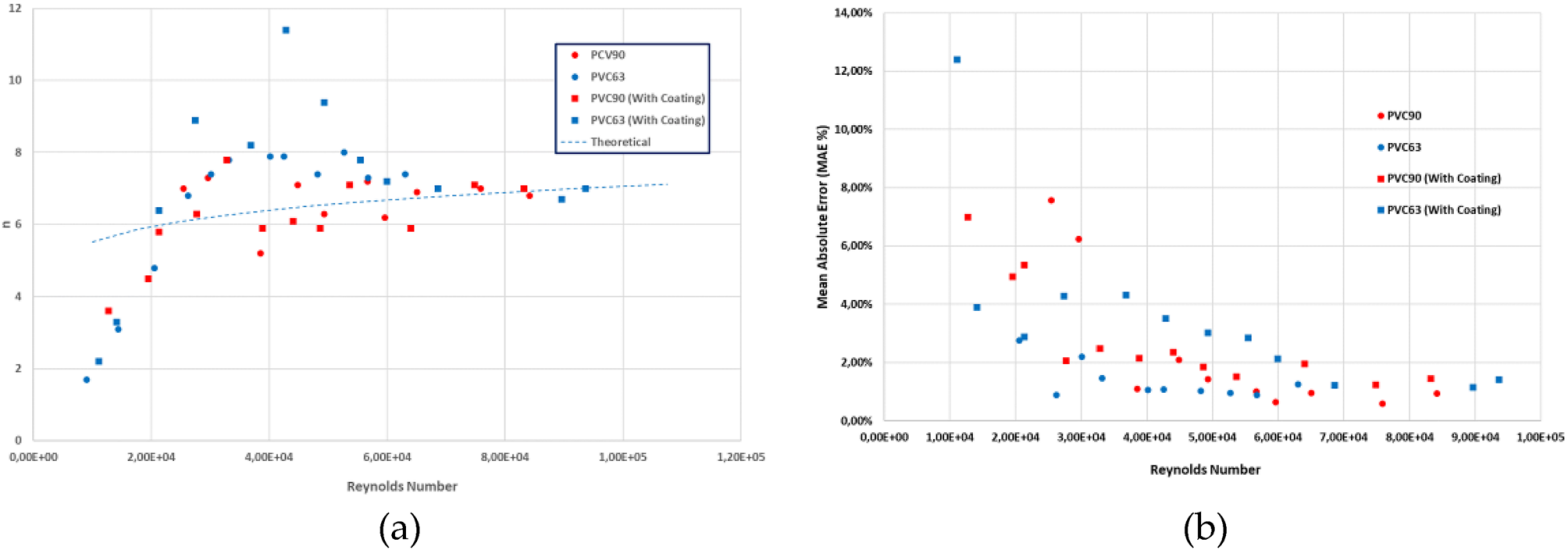
Comparison of the fitted *n* parameter of the Power-Law model for both treated and untreated pipes (**a**), comparison of Mean Absolute Errors of the Power-Law fitting for the treated and untreated pipes (**b**).

Figure 13 shows the comparison between coated and uncoated pipes in terms of the parameter *n* value of the Power-Law model (a) and in terms of the MAE (b) of the fitting to the Power-Law model. These graphs show that no significant differences were found either in the MAE or in the *n* value between treated and untreated pipes. This observation seems to indicate that velocity profiles are similar in both treated and untreated pipes, given that the *n* value directly corresponds to the slope of the turbulent velocity profile. Consequently, it can be inferred that the proposed models might also be valid to describe the velocity profiles within hydrophobic pipes.

Although no significant differences were found in the outer turbulent layer, according to the theories that explains the hydrophobic effect, these flow velocity differences must be restricted to the viscous sublayer or the overlap regions. These regions could not be explored in this experiment.

## Conclusions

An experiment was carried out to analyze a hydrophobic coating applied to the inner wall of a pipe and its effect on the head losses or Drag Reduction Percentage (%DR) of said pipe and the velocity profile.

This experiment demonstrated that, for relatively low Reynolds numbers, the measured head losses are considerably lower in treated pipes than in non-treated ones. The %DR decreases for increasing Reynolds number (Re) values and tends asymptotically to zero for high Re values for high turbulent flows. This may be explained by considering a partial slip condition caused by the hydrophobic coating, particularly notable in relatively low turbulent flows. This effect becomes negligible for flows with a greater degree of turbulence.

Because of the %DR, the friction factor for hydrophobic pipes lies below the hydraulically smooth pipes curve and growths for increasing Re until it tends to follow this curve for high Re values, meaning that the treated pipe shows similar behavior to the hydraulically smooth one.

No significant differences that explain this head loss reduction were found in the velocity profiles of the outer turbulent layer. These differences might be limited to the viscous sublayer and to the overlap region since these specific areas could not be adequately explored in this experiment.

As a general conclusion, the application of a hydrophobic product to the inner wall of the pipe under the described hydraulic regime can considerably reduce the energy required to transport a fluid in a pressurized pipe. Furthermore, this enhancement in energy efficiency can aid in reducing CO_2_ emissions, thus contributing to achieving the Sustainable Development Goals (SDG) proposed by the United Nations in the 2030 agenda.

This work opens a new research line focused on the application of a hydrophobic coating to reduce head losses and improve energy use efficiency in fluid transport through pressurized pipes. The following are some suggestions for further research regarding this line:

- To extend the experimental tests to other pipe materials.
- To use precise velocity measuring techniques to analyze the fluid flow in the near-wall regions.
- To analyze the energy reduction potential of multiphase flows. The multiphase flow caused by the injection of air bubbles that may contribute to increasing the hydrophobic effect could be of great interest.

## Data Availability

The data and materials will be available under request to the corresponding author (email: jreca@ual.es).
